# Knowledge, attitude, and practice of healthcare professionals toward clinically applying graduated compression stockings: results of a Chinese web-based survey

**DOI:** 10.1007/s11239-018-1749-4

**Published:** 2018-10-06

**Authors:** Yaping Xu, Wei Wang, Jing Zhao, Jiahuan Wang, Tingting Zhao

**Affiliations:** 1 Department of Orthopedics, China-Japan Friendship Hospital, Beijing, China; 2 Bone Necrosis and Joint Preservation Reconstruction Center, China-Japan Friendship Hospital, Beijing, China; 3 Department of Nursing, China-Japan Friendship Hospital, Beijing, China; 4 Department of Bone and Joint Surgery, China-Japan Friendship Hospital, 2 Yinghua Dongjie, Hepingli, Chaoyang District, Beijing, 100029 China

**Keywords:** Knowledge, Attitude, Practice, Graduated compression stockings

## Abstract

This study had three objectives: (1) to investigate healthcare professionals’ knowledge, attitude, and practice toward clinically applying graduated compression stockings, (2) to analyze the influencing factors, and (3) to provide data to support departments that develop health policies. A self-administered web-based survey was completed by 1,444 healthcare professionals, including physicians and nurses from 15tertiary hospitals and four secondary hospitals from 10 provinces in China. Reliability analysis and exploratory factor analysis were used to evaluate the researcher-designed questionnaire’s reliability and validity. The formal questionnaire, which included demographic data (eight items), knowledge (ten items), attitudes (four items), and clinical practice patterns (six items), was distributed among healthcare professionals. The relationships and mechanisms among the variables were explored using descriptive statistical analysis, Pearson’s correlation coefficients, and multiple linear regression analysis. Of the 1,444 respondents, 31.2% had good knowledge of clinically applying graduated compression stockings, 83.5% had a positive attitude toward clinically applying them, and 30.4% of respondents exhibited normative behavior when applying them. The multiple linear regression analysis indicated that training was an important factor influencing the knowledge, attitude, and practice toward clinically applying graduated compression stockings. The healthcare professionals’ attitude toward clinically applying graduated compression stockings was positive, but the related knowledge was poor, and the code of behavior was deficient. Medical institutions should improve training for clinically applying graduated compression stockings among healthcare professionals and standardize the use of graduated compression stockings to facilitate the prevention of hospital-acquired venous thromboembolism.

## Highlights

- This is the first study to investigate healthcare professionals’ knowledge, attitude, and practice regarding the clinical application of graduated compression stockings in China.
- The healthcare professionals’ attitude towards clinically applying graduated compression stockings on patients was positive; however, our assessment demonstrated that gaps in knowledge and behavior existed.
- Educational programs for medical professionals that improve knowledge and practice regarding the use of graduated compression stockings may aid in the prevention of venous thromboembolism.

## Introduction

Venous thromboembolism (VTE), which includes deep vein thrombosis (DVT) and pulmonary embolism (PE), is a serious condition that affects patients’ outcomes. Lower extremity DVT can result in death or significant disability due to PE or post-thrombotic syndrome (PTS). VTE is the third leading cause of cardiovascular-associated deaths worldwide [1].

The Korean Practice Guidelines [2] recommend that compression stockings are used to reduce the risk of PTS in patients with lower extremity DVT (Class IIa, Level A). The American College of Chest Physicians released the 10th edition of the Antithrombotic Guidelines [3] (AT10) in 2016. The AT10 states that patients with acute DVT of the leg should not use compression stockings routinely to prevent PTS (Grade 2B), but for patients with acute or chronic symptoms, a trial of graduated compression stockings (GCS) is often justified. Compression therapy is a frequently used physical therapy in conditions involving venous and lymphatic insufficiency in the lower limbs, including varicosities, lymphedema, venous eczema and ulceration, and DVT and PTS [4]. If an inpatient is assessed into the high-risk hierarchy for VTE, GCS is recommended as a physical preventive measure in the China-Japan Friendship Hospital Health Alliance. However, some scholars have questioned GCS’ effect in recent years [5]. There is a Chinese saying that “If a man will do good, he will first need to gain his tools.” In clinical practice, we have anecdotally found patients’ use of GCS depends largely on the information provided by healthcare professionals. Thus, our research team asked the questions, “how can we expect patients to be provided with good information if the level of healthcare professionals’ KAP of GCS is not high? Further, if patients are unable to use GCS properly, how can we expect GCS to work as a treatment for DVT?” which highlights the need to understand the knowledge, attitudes, and behaviors (KAP) of practitioners who are using GCS as a treatment. Meanwhile, it is necessary to pay attention to the correct use of GCS when evaluating its effects.

GCS work by exerting the greatest degree of compression at the ankle, with the level of compression gradually decreasing up the garment; this pressure gradient ensures that blood flows upward toward the heart instead of refluxing downward to the foot or laterally into the superficial veins [4]. Applying GCS properly is a prerequisite for their function. Inadequate on-the-job training of healthcare professionals might lead them to use the GCS improperly. However, there is a paucity of research that has focused on healthcare professionals’ KAP toward clinically applying GCS. To fill this gap, this study aimed to investigate their KAP toward clinically applying GCS and to provide data to support departments that create health policies.

## Methods

### Survey design

This was a non-interventional, anonymized, self-administered, one-time web-based survey of healthcare professionals in China. This survey was conducted from February 21 to March 2, 2018. The study was approved by the institutional review board of the China-Japan Friendship Hospital and adhered to the Helsinki Declaration. Written informed consent was not required because the questionnaire survey of the employees was anonymous and had less than minimal risk.

### Survey sample

The sample consisted of 1444 healthcare professionals, including physicians and nurses, who had obtained the practice qualification and registration of the People’s Republic of China and engaged in medical care work for more than one year. Of these, 130 participants took part in a preliminary experiment to test the reliability and validity of a questionnaire that was designed by experts from the China-Japan Friendship Hospital. The sampled hospitals included 15 tertiary hospitals and four secondary hospitals from 10 provinces in China. In some tertiary hospitals, doctors and nurses provide different types of consultations. To some extent, nurses assess the risk of thrombosis in patients and provide GCS based on risk stratification. However, in clinical practice, physicians also focus on how to apply and manage the stockings in China. Thus, both doctors and nurses have an obligation to deliver health messages to patients. For these reasons, we elected not to separate physicians and nurses in our sample.

### Survey questionnaire

The questionnaire items were designed by experts from the working group for the prevention and treatment of VTE of the China-Japan Friendship Hospital. An original questionnaire that included 32 items was developed based on a preliminary survey of 130 cases to evaluate its reliability and validity. In the pre-investigation, researchers screened all items and created the formal questionnaire through exploratory factor analysis (EFA) using IBM SPSS Statistics for Windows version 19.0 (IBM Corp., Armonk, NY, USA). Finally, a 28-item validated questionnaire was used to collect demographic data (eight items), knowledge (ten items), attitudes (four items), and clinical practice patterns (six items). The Cronbach’s alphas for the knowledge, attitudes, and clinical practice pattern scales were 0.949, 0.944, and 0.911 respectively. The overall scale for KAP had a high Cronbach’s alpha coefficient (0.943).

The demographic characteristics included sex, age, service years, hospital level, profession, highest education attained, professional title, and GCS application training. The questionnaire did not ask for any personally identifying information. The other 20 items were single choice questions, which were scored on a 5-point Likert scale. The 10 items on the knowledge dimension were assessed using a 5-point Likert scale ranging from 1 to 5 (1 = very unfamiliar, 2 = unfamiliar, 3 = generally familiar, 4 = familiar, and 5 = very familiar). Higher scores represented better knowledge. The four items on the attitude dimension were evaluated on a 5-point Likert scale ranging from 1 to 5 (1 = disagree strongly, 2 = disagree, 3 = generally agree, 4 = agree, and 5 = agree strongly). Higher scores indicated a more positive attitude. The six items on the practice dimension were scored on a 5-point Likert scale ranging from 1 to 5 (1 = never, 2 = seldom, 3 = occasionally, 4 = often, and 5 = frequently). Higher scores represent more normative behavior.

### Survey procedure

The survey was a closed survey. The researchers uploaded the questionnaire to So Jump (https://www.wjx.cn/) and generated a QR code. The QR code was then distributed to healthcare professionals through the platform of the China-Japan Friendship Hospital Health Alliance. Participants identified the QR code through WeChat (Tencent Holdings, Shenzhen, China) and responded.

The survey had two steps. First, participants were asked to report their demographic characteristics. Second, participants completed the survey about KAP toward clinically applying GCS. The questionnaire was designed in such a way that it could not be submitted until all questions had been answered. Participants could review and change their responses before submission. The survey period lasted for 10 days, and during this time, 1,444 healthcare professionals responded effectively to the survey.

### Statistical analysis

All analyses of the data were performed using IBM SPSS Statistics for Windows version 19.0 (IBM Corp., Armonk, NY, USA). Reliability analysis and EFA were used to evaluate the researcher-designed questionnaire’s reliability and validity. Descriptive statistical analysis was used to summarize the 1,444 healthcare professionals’ demographic characteristics. Continuous data were expressed as means and standard deviations (*M* ± *SD*), and comparisons were made using the Student’s *t*-test. Categorical data were reported as absolute numbers and proportions, and comparisons were made using the chi-squared test. Pearson’s correlation coefficients were computed to examine the relationships between KAP toward clinically applying GCS. Multiple regression analysis was used to analyze the factors influencing the KAP of healthcare professionals toward clinically applying GCS. The adjusted *R*^2^ and 95% confidence intervals were calculated. A two-tailed *p*-value of < 0.05 was considered statistically significant.

## Results

### Descriptive results

The demographic characteristics are shown in Table 1. The data indicated that most of the sample was nurses (81.9%). Among the participants surveyed, 82.4% were from a tertiary hospital, 76.2% had a bachelor’s degree or above, and 67.9% had not received training for applying GCS.

**Table 1:** Characteristics of the respondents (*N* = 1,444)

Characteristic	Categories	*n*	%
Sex	Male	214	14.8
	Female	1230	85.2
Age (years)	< 20	5	0.4
	20–29	519	35.9
	30–39	601	41.6
	≥ 40	319	22.1
Service years	1–5	450	31.2
	6–10	377	26.1
	11–19	324	22.4
	≥ 20	293	20.3
Hospital level	Tertiary hospital A	955	66.1
	Tertiary hospital B	91	6.3
	Secondary hospital A	390	27
	Secondary hospital B	8	0.6
Profession	Doctor	261	18.1
	Nurse	1183	81.9
Highest education attained	Secondary^a^	27	1.9
	College	316	21.9
	Bachelor’s degree	943	65.3
	Master’s degree or above	158	10.9
Professional title	Junior	764	52.9
	Intermediate	535	37.1
	Senior	145	10
GCS application training	Yes	463	32.1
	No	981	67.9

*GCS* graduated compression stockings

^a^Even in some Tertiary hospitals in China, there are a few nurses who have worked for more than 20 years who have only completed secondary education

The means, *SD*, and Pearson’s correlation coefficients of the continuous variables are shown in Table 2. As the results indicate, all variables were significantly correlated with each other. There was a significant positive and weak correlation between knowledge and attitude (*r* = 0.316, *p* < 0.01). There was a significant strong and positive correlation between knowledge and practice (*r* = 0.653, *p* < 0.01), and a significant positive and weak correlation between attitude and behavior (*r* = 0.330, *p* < 0.01).

**Table 2:** The means, standard deviations, and Pearson’s correlation coefficients (*N* = 1,444)

Variables	*M*	*SD*	Minimum	Maximum	Knowledge	Attitude	Behavior
Knowledge	30.12	8.16	10	50	1		
Attitude	16.50	2.63	6	30	0.316*	1	
Behavior	16.94	5.81	4	20	0.653*	0.330*	1

The correlation is significant at the 0.01 level (two-tailed)

*M* mean, *SD* standard deviation

*p < 0.01

**Fig. 1:**
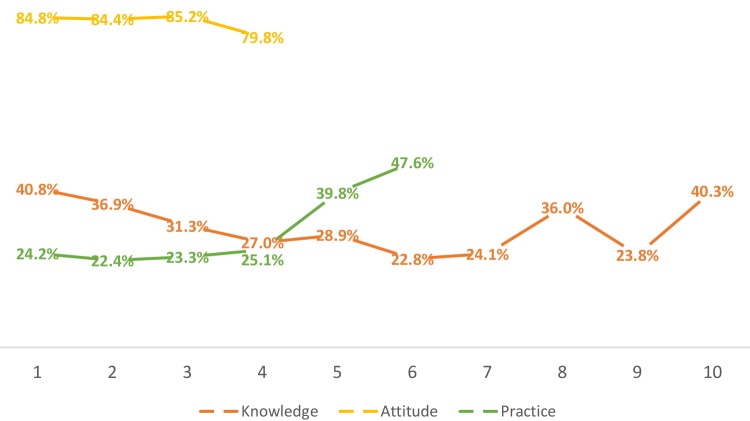
Proportion of the classification of “good” for the 20 items of the knowledge, attitude, and practice dimensions (*N* = 1444). *Knowledge 1* Maintenance instructions, *Knowledge 2* Washing method, *Knowledge 3* Wearing method, *Knowledge 4* Service life, *Knowledge 5* Timing, *Knowledge 6* Size, *Knowledge 7* Length, *Knowledge 8* Contraindications, *Knowledge 9* Pressure level, *Knowledge 10* Indications. *Attitude 1* The benefits of graduated compression stockings (GCS) should be actively communicated to patients and their caregiver, *Attitude 2* Health education about GCS should be strengthened for patients and their caregiver, *Attitude 3* Medical institutions and managers should pay attention to GCS application training for healthcare professionals, *Attitude 4* The use of GCS should be recommended to patients. *Practice 1* I fully inform the patient and their caregiver about the instructions for using GCS, *Practice 2* I actively inform patients and their caregiver about the benefits of using GCS, *Practice 3* I have actively participated in training for the use of GCS, *Practice 4* I check if the patient is using GCS properly from time to time, *Practice 5* My work using GCS is established, *Practice 6* In clinical practice, the medical staffs’ work using GCS is established

For the 10 items of the knowledge dimension, reports of “very familiar” and “familiar” were classified as the participant being qualified in clinically applying GCS. The survey showed that 31.2% of participants had good knowledge of GCS. In response to the four items of the attitude dimension, " agree strongly” and “agree” were attributed as being positive attitudes. In addition, 83.5% of the participants were active in clinically applying GCS. For the six items of the behavior dimension, “always” and “often” were considered to be normative behavior. It was found that 30.4% of the healthcare professionals’ behavior was normative. On the 5-point Likert scales, a score of 4 or 5 was classified as “good” by the researchers. Figure 1 shows the proportion of the 24 items that were classified as good in the three dimensions.

### Stepwise linear regression model

Several stepwise linear regression analyses were performed to analyze the factors influencing the respondents’ KAP toward clinically applying GCS (Table 3). The results showed that GCS application training was an important factor influencing their KAP.

**Table 3:** Stepwise linear regression analysis of the knowledge, attitude, and practice dimensions (*n* = 1,444)

	Total score of the knowledge dimension	Total score of the attitude dimension	Total score of the behavior dimension
GCS application training			
*B*	− 8.392***	− 1.064***	− 5.376***
Std. Error	0.402	0.145	0.296
95% CI for *B*	− 9.180, − 7.604	− 1.349, − 0.778	− .957, − 4.795
(Lower bound/upper bound)			
Beta	− 0.48	− 0.189	− 0.432
Highest education			
*B*	− 1.161***	–	− 0.612**
Std. Error	0.314	–	0.229
95% CI for *B*	− 1.776, − 0.546	–	− 1.060, − 0.163
(Lower bound/upper bound)			
Beta	− 0.088	–	− 0.065
Service years			
*B*	0.492**	–	–
Std. Error	0.169	–	–
95% CI for B	0.159, -0.824	–	–
(Lower bound/upper bound)			
Beta	0.067	–	–
Sex^a^			
*B*	− 1.378*	–	− 1.654***
Std. Error	0.54	–	0.398
95% CI for *B*	− 2.437, − 0.318	–	− 2.433, − 0.874
(Lower bound/upper bound)			
Beta	− 0.06	–	− 0.101
Hospital level			
*B*	–	0.417***	0.845***
Std. Error	–	0.076	0.156
95% CI for *B*	–	0.269, 0.566	0.539, 1.150
(Lower bound/upper bound)			
Beta	–	0.143	0.131
Constant			
*B*	48.936***	16.955***	29.403***
Std. Error	1.631	0.318	1.233
95% CI for *B*	45.736, 52.137	16.332, 17.579	26.984, 31.822
(Lower bound/upper bound)			
Adjusted *R*^2^	0.244	0.057	0.206
*p*	0	0	0
Std. error of the estimate	7.09	2.55	5.17
*N*	1444	1444	1444

*GCS* graduated compression stockings, *Std. error* standard error, *CI* confidence interval.

*p < 0.05; **p < 0.01; ***p < 0.001.

^a^Male sex was associated with higher scores for knowledge and behavior dimensions; 14.8% of participants were male and most were physicians with a high level of education.

## Discussion

### The healthcare professionals’ level of knowledge of clinically applying GCS

The results of this study showed that the overall mean score of the knowledge of clinically applying GCS was 30.12 (± 8.16) out of a possible score of 50. For the 10 items on the knowledge dimension, reports of being “very familiar” and “familiar” were classified as the participant being qualified in clinically applying GCS. Only 31.2% of participants had good knowledge of GCS and the percentage of participants who had a good grasp of each item was less than one-third, which further suggests that the knowledge level of clinically applying GCS among medical staff is low. The survey results of Tang et al. [6] showed that only 10.3% of the medical staff knew the appropriate indications for medical VTE prophylaxis (e.g., GCS and intermittent pneumatic compression). As Fig. 1 showed, 40.3% of participants knew the indications for GCS, and 36.0% of participants knew the contraindications for GCS. The study of Oh, Boo, and Lee [7] also stated that the mean score was 3.39 (± 0.95) out of a possible score of 5 for effective use of mechanical devices for VTE prevention (e.g., GCS, intermittent pneumatic compression, or a foot pump). All these suggested healthcare professionals’ knowledge of clinically applying GCS was deficient to some extent. The low knowledge level of clinically applying GCS among healthcare professionals deserves attention and should be improved. The healthcare professionals’ knowledge level directly affects the effectiveness of clinical practice. In this survey, 67.9% of participants had no training in applying GCS. Medical institutions and departmental managers should provide training to increase the knowledge of medical staff about the application of GCS and to improve the quality of clinical practice.

### The attitude of healthcare professionals regarding the use of GCS

This investigation showed that the overall mean score of the attitude toward clinically applying GCS was 16.50 (± 2.63) out of a possible score of 20, and 83.5% of the respondents had a positive attitude toward clinically applying GCS. This finding might be related to the policy issued by the National Health and Family Planning Commission of the People’s Republic of China. Preventing DVT has been emphasized in the management of a single disease, [8] and the use of GCS is physical prevention against DVT.

### The practice of the healthcare professionals regarding the use of GCS

The survey found that the overall mean score of the practice of clinically applying GCS was 16.94 (± 5.81) out of a possible score of 30, and only 30.4% of the medical staffs’ behavior when applying GCS was standardized, which might be due to the low level of knowledge and lack of training. Many studies [7, 9–11] have shown that nurses lack VTE training and their behavior is not regulated. The majority of the sample were nurses (81.9%), which is consistent with previous studies. The strong positive correlation between knowledge and behavior suggests the importance and necessity of training.

### The relationships between the KAP toward clinically applying GCS

The study showed that the knowledge and attitude of the healthcare professionals toward clinically applying GCS were positively and weakly correlated, their knowledge and behavior were strongly and positively correlated, and their attitude and behavior were positively and weakly correlated. These findings indicate that increasing the knowledge level is beneficial to the overall improvement of the healthcare professionals’ KAP toward clinically applying GCS. The survey showed that only 32.1% of participants had received training related to GCS, which is consistent with a previous study [7, 9–11]. The relatively lower rate of GCS in-service education may be related to Chinese healthcare policies. Several stepwise linear regression analyses showed that GCS application training was an important factor influencing the KAP of healthcare professionals. Once medical institutions and management departments pay enough attention to training, effectively implement relevant training, and standardize training, the improvement of the knowledge level might be very easy to achieve. Furthermore, the level of the healthcare professionals’ KAP toward clinically applying GCS might be greatly improved, which lays a good foundation for the prevention and treatment of VTE.

## Conclusion

This study focused on healthcare professionals’ KAP toward clinically applying GCS. It is the first study to investigate healthcare professionals’ KAP toward clinically applying GCS in China. We hope that this study will prompt researchers to pay attention to the accuracy of GCS use when evaluating its effects, as the correct use of GCS cannot be separated from the health education of healthcare professionals. Our findings indicated that the level of healthcare professionals’ KAP regarding GCS is of concern. The results indicate the importance of in-service training and they provide data to support departments that formulate health policies. The proper use of GCS deserves attention. In the future, we should work on how best to develop training programs within the hospital setting and hope to identify which professional group would benefit most from such training.

## Data Availability

Data collected from the survey were anonymized. The raw data from which the paper’s results were derived can be made available on request.
